# Phospholipase C-β4 Is Essential for the Progression of the Normal Sleep Sequence and Ultradian Body Temperature Rhythms in Mice

**DOI:** 10.1371/journal.pone.0007737

**Published:** 2009-11-09

**Authors:** Masayuki Ikeda, Moritoshi Hirono, Takashi Sugiyama, Takahiro Moriya, Masami Ikeda-Sagara, Naomi Eguchi, Yoshihiro Urade, Tohru Yoshioka

**Affiliations:** 1 Department of Chronobiology, Graduate School of Innovative Life Science, University of Toyama, Toyama, Japan; 2 Department of Molecular Neurobiology, Advanced Institute for Biological Science, Waseda University, Tokyo, Japan; 3 Department of Molecular Behavioral Biology, Osaka Bioscience Institute, Osaka, Japan; 4 Yamada Research Unit, RIKEN Brain Science Institute, Saitama, Japan; 5 Department of Cellular Signaling, Graduate School of Pharmaceutical Sciences, Tohoku University, Sendai, Japan; 6 Center of Excellent for Environmental Medicine, Graduate Institute of Medical Sciences, Kaohsiung Medical University, Kaohsiung, Taiwan; Vanderbilt University, United States of America

## Abstract

**Background:**

The sleep sequence: i) non-REM sleep, ii) REM sleep, and iii) wakefulness, is stable and widely preserved in mammals, but the underlying mechanisms are unknown. It has been shown that this sequence is disrupted by sudden REM sleep onset during active wakefulness (i.e., narcolepsy) in orexin-deficient mutant animals. Phospholipase C (PLC) mediates the signaling of numerous metabotropic receptors, including orexin receptors. Among the several PLC subtypes, the β4 subtype is uniquely localized in the geniculate nucleus of thalamus which is hypothesized to have a critical role in the transition and maintenance of sleep stages. In fact, we have reported irregular theta wave frequency during REM sleep in PLC-β4-deficient mutant (PLC-β4−/−) mice. Daily behavioral phenotypes and metabotropic receptors involved have not been analyzed in detail in PLC-β4−/− mice, however.

**Methodology/Principal Findings:**

Therefore, we analyzed 24-h sleep electroencephalogram in PLC-β4−/− mice. PLC-β4−/− mice exhibited normal non-REM sleep both during the day and nighttime. PLC-β4−/− mice, however, exhibited increased REM sleep during the night, their active period. Also, their sleep was fragmented with unusual wake-to-REM sleep transitions, both during the day and nighttime. In addition, PLC-β4−/− mice reduced ultradian body temperature rhythms and elevated body temperatures during the daytime, but had normal homeothermal response to acute shifts in ambient temperatures (22°C–4°C). Within the most likely brain areas to produce these behavioral phenotypes, we found that, not orexin, but group-1 metabotropic glutamate receptor (mGluR)-mediated Ca^2+^ mobilization was significantly reduced in the dorsal lateral geniculate nucleus (LGNd) of PLC-β4−/− mice. Voltage clamp recordings revealed that group-1 mGluR-mediated currents in LGNd relay neurons (inward in wild-type mice) were outward in PLC-β4−/− mice.

**Conclusions/Significance:**

These lines of evidence indicate that impaired LGNd relay, possibly mediated via group-1 mGluR, may underlie irregular sleep sequences and ultradian body temperature rhythms in PLC-β4−/− mice.

## Introduction

The behavioral state of sleep consists of two basic stages: (1) rapid-eye-movement (REM) sleep with typical theta electroencephalogram (EEG) waves and (2) restful non-REM sleep, with slow EEG waves. The sleep sequence: i) non-REM sleep, ii) REM sleep, and iii) wakefulness, is stable and widely preserved in mammals, with REM sleep consistently following non-REM sleep. While understanding of the REM-regulating network remains incomplete, it is known that in general, the occurrence of REM sleep follows the generation of ponto-geniculo-occipital waves that arise in the pons and are transmitted to the thalamic lateral geniculate nucleus (LGN) and visual occipital cortex. The REM-On neurons in the laterodorsal and pedunculopontine tegmental nuclei under the regulation of serotonergic dorsal raphe neurons and noradrenergic locus coeruleus neurons may be critical for the activation of the pontine reticular formation (PRF) and ultimately contribute to the shift to REM sleep [1], [2]. However, understanding of the REM-regulating network is incomplete due to the large network size.

Narcolepsy is characterized by sudden REM sleep attacks during active wakefulness, and thus represents a rare condition in which the preserved sleep sequence is disrupted. Orexin receptor mutations are the cause of canine narcolepsy [3], and orexin knockout mice displayed behaviors resembling aspects of narcolepsy [4]–[6], suggesting that orexinergic neuronal transmission is an essential component of the preserved sleep sequence. Orexin may regulate REM sleep within the PRF since perfusion of antisense oligonucleotides for the orexin-2 receptor into the PRF produces irregular REM sleep similar to that of orexin knockout mice [7]. On the other hand, orexin receptive neurons are densely distributed in the locus coeruleus [8] and thalamic rhomboid and centromedial nuclei [9], [10], where they may receive projections from the PRF. There are multiple and diverse orexinergic projections in the brain [11], and the specific orexin projection responsible for the disruption of the sleep sequence in orexin-deficient mutant animals has not been elucidated.

Phospholipase C (PLC) is an enzyme that mediates cellular signaling via various metabotropic receptors. Orexin-1 and -2 receptors are metabotropic receptors, presumably linked to PLC [12]. To date, several PLC subtypes have been cloned and the β4 subtype is located in the cerebellum [13], [14] and visual input pathways such as retina [15], superior colliculus, and thalamic geniculate nucleus [16]. The thalamic geniculate nucleus is also part of the sleep-regulating system, and exhibits robust expression of PLC-β4, especially in the dorsal LGN (LGNd)[16], where pontomedullary sleep-regulating neurons terminate. LGNd relay neurons are also part of a corticothalamic feedback loop, which is hypothesized to have a critical role in the transition of sleep stages [17]–[19]. We have reported irregular EEG power spectrum during REM sleep in PLC-β4-deficient mutant (PLC-β4−/−) mice when it was analyzed randomly from their sleep EEG [16]. Here, we further investigate possible roles of PLC-β4 in daily sleep-wake and body temperature rhythms.

## Materials and Methods

### Sleep Recording

Methods for the creation of PLC-β4−/− mice were described previously [13]. Also, sleep-wake stages of mice were analyzed based on EEG and electromyogram (EMG) as described previously [20]. Briefly, 12–15 weeks old male wild-type C57BL6J mice (*n* = 6) and PLC-β4−/− mice (*n* = 6) were used in the experiments. The PLC-β4−/− mice were littermates intercrossed between male and female heterozygotes that were backcrossed to C57BL6J mice for at least seven generations. The animals were maintained under a 12-hour light/dark cycle in sound-attenuated temperature-controlled (22.0±1.0°C) chambers. Mice were deeply anesthetized with pentobarbital (50 mg/kg, i.p.) and implanted with two EEG electrodes over the parietal cortex and the cerebellum. Two EMG electrodes were inserted in the muscle of the dorsal neck. Animals were allowed at least 10 days of recovery from surgery, and after habituation to experimental conditions, three consecutive 24-hour recordings.

The vigilance states were automatically classified off-line by 4-second epochs into three stages of wake, non-REM, and REM sleep by SLEEPSIGN (Kissei COMTEC, Matsumoto, Japan), according to the standard criteria [20], [21]. As a final step, defined sleep–wake stages were examined visually, and corrected, if necessary. The sleep scoring data on the second day were used for further statistical comparisons.

### Measurement of Body Temperature Rhythms

Animals were anesthetized by an intraperitoneal injection of pentobarbital (50 mg/kg, i.p.) for abdominal surgery. A telemetry probe was implanted intraperitoneally in wild type mice (n = 5) and PLC-β4−/− mice (n = 5). After 10 days recovery, the body temperature was continuously monitored at 5 minute intervals for 3 weeks using a telemetry system (Dataquest LabPRO ver. 3.10, Data Sciences International, St. Paul, MN). Daily variations in body temperature levels were averaged from data recorded for three consecutive days on the second week of monitoring. During the recording period, food and water were given *ad libitum* and mice were kept under a 12-h light/dark cycle at 22.0±1.0°C. To analyze homeostatic thermal regulation, wild-type (n = 4) and PLC-β4−/− mice (n = 4) were transferred for 2 hours to a cage with a reduced temperature (4.0±1.0°C) during the light period. The above *in vivo* recording experiments were designed to minimize the number of animals used and their suffering based on international guidelines and were approved by the appropriate animal care and use committees at Osaka Bioscience Institute and Waseda University.

### Immunocytochemistry

Affinity-purified rabbit polyclonal antibodies for PLC-β1, -β2, -β3 and -β4 (Santa Cruz Biotechnology, Santa Cruz, CA) were used. One month old mice were anesthetized with pentobarbital and transcardially perfused with 4% phosphate-buffered paraformaldehyde (4°C; pH 7.4). The brains were immersed overnight in the fixative and embedded in paraffin and sectioned at 4 µm thick. As a blocking step, the sections were incubated with 3% H_2_O_2_ distilled water for 10 minutes, and 10% normal goat serum for 1 hour. The antibodies against PLC-β1 (1/500), PLC-β2 (1/500), PLC-β3 (1/100) or PLC-β4 (1/50) were applied to sections overnight at 4°C. Subsequently, sections were incubated with biotin-conjugated goat anti-rabbit IgG (Vector Laboratories, Burlingame, CA) for 1 hour at room temperature. Sections were then incubated with peroxidase-conjugated streptoavidin (Nichirei, Tokyo, Japan) for 1 hour at room temperature. Between each incubation step the sections were rinsed twice in 0.01 M phosphate-buffered saline (pH 7.4) for 5 minutes. The final peroxidase reaction was performed using 0.05% diaminobenzidine and 0.005% H_2_O_2_. The same sections were also stained with cresyl violet using conventional methods.

### Ca^2+^ Imaging

Macroscopic Ca^2+^ imaging was performed as described previously [22]. Briefly, coronal slices (200 µm) containing the geniculate nuclei and hippocampus were prepared using a vibrating blade microtome in ice-cold high-Mg^2+^ (4 mM) and low-Ca^2+^ (0.5 mM) artificial cerebrospinal fluid (ACSF) bubbled with 95% O_2_/5% CO_2_. The slices were incubated for 3–5 hours in regular ACSF (2.5 mM CaCl_2_ and 1 mM MgCl_2_) bubbled with 95% O_2_/5% CO_2_. For Ca^2+^ imaging, the slices were immersed for 1 hour in regular ACSF containing 10 µM fura-2 AM (Molecular Probes, Eugene, OR) and 0.001% Cremophore El (Sigma). After washing out the fura-2 AM solution with ACSF, slices were placed in a glass-bottom chamber for optical measurements. Images were obtained at 10-second intervals using an upright microscope (Axioplan2; Carl Zeiss, Thornwood, NY). The intensity ratio in the region of interest was analyzed using a digital imaging system (Hamamatsu Photonics, Hamamatsu, Japan). (*RS*)-3,5-dihydroxy-phenylglycine (DHPG), 1*S*,3*R*-1-aminocyclopentane-1,3-dicarboxylic acid (1*S*, 3*R*-ACPD), orexin A, or orexin B (Sigma-RBI, St. Louis, MO) was applied for 1 minute by switching the perfusate (regular ACSF supplemented with 1 µM tetrodotoxin). The 50 mM K^+^ ACSF was perfused for 45 seconds to estimate the maximal Ca^2+^ response.

### Electrophysiology

Whole-cell voltage-clamp recordings were made from relay neurons (cell body diameter = 15–25 µm)[23] and interneurons (≅ 10 µm)[24] in LGNd slices under Nomarski optics using a water-immersion objective (Achroplan 63×/0.90 w, Carl Zeiss). Patch pipettes (3–5 MΩ) were filled with intracellular solution containing 150 mM CsCH_3_SO_3_, 5 mM KCl, 0.1 mM K-EGTA, 5.0 mM Na-HEPES, 3.0 mM Mg-ATP, and 0.4 mM Na-GTP (pH 7.4). Membrane currents were recorded using an EPC-7 amplifier (List Electronic, Darmstadt, Germany) and pCLAMP software (Molecular Devices, Sunnyvale, CA), digitized, and stored on a diskette for later analysis. The series resistance compensation control of the amplifier was set at 50–70%. DHPG and α-amino-3-hydroxy-5-methyl-4-isoxazolepropionic acid (AMPA, Sigma-RBI) were applied in the vicinity of neurons via a Y-tube circulating regular ACSF supplemented with 1 µM tetrodotoxin. All experiments were performed at room temperature. Further details were described previously [13]. The above *in vitro* recording experiments were approved by the appropriate animal care and use committees at Waseda University and University of Toyama.

### Statistical Analysis

All data are presented as means with standard errors. A two-tailed Student's *t*-test was used for the genotype-wise comparisons.

## Results

### Sleep Abnormality in PLC-β4−/− Mice

**Figure 1:**
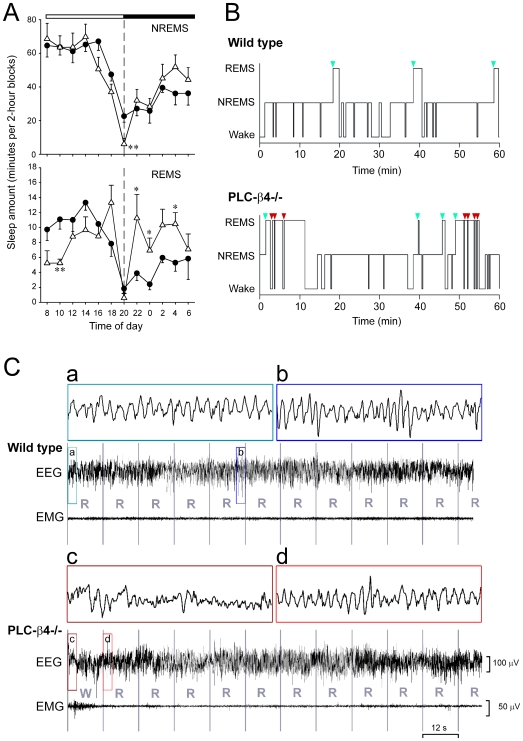
Sleep abnormality in PLC-β4−/− mice. A. The time courses of non-REM sleep (NREMS) and REM sleep (REMS) were plotted in 2 hour intervals for wild-type mice (n = 6, closed circles) and PLC-β4−/− mice (n = 6, open triangles). There was no difference in the time course of non-REM sleep except for a significant loss of sleep at the onset of darkness in PLC-β4−/− mice. The circadian time course of REM sleep was eliminated in PLC-β4−/− mice. *P<.05; **P<.01 compared with the corresponding wild-type group by Student's *t*-test. B. Hypnographic analysis also indicated abnormal REM sleep episodes in PLC-β4−/− mice. REM sleep regularly occurred after non-REM sleep in wild-type mice (the regular onset of REM sleep is marked by green arrows) whereas REM sleep episodes with frequent bouts of wakefulness were observed in PLC-β4−/− mice (REM sleep onset after wakefulness is marked by red arrows). C. *Upper*. An example sleep polygraph (combination of EEG and EMG) during regular REM sleep in a wild type mouse. *Lower*. An example sleep polygraph showing direct REM sleep transition from wakefulness in a PLC-β4−/− mouse. Squared 3-second EEG waves (a–d) were enlarged on the top. Typical theta waves were observed during both regular (a,b) and irregular (d) REM sleep. Estimated sleep stages are presented in 12-second bins. W: wakefulness, R: REM sleep.

The length of non-REM sleep in PLC-β4−/− mice (352.9±14.9 minutes during the day and 207.7±29.9 minutes during the night, n = 6) was not significantly different from that in wild-type mice (365.4±19.0 minutes during the day and 187.5±15.7 minutes during the night, n = 6, n.s. by Student's *t*-test). Also, both genotypes exhibited similar delta (0.75–4.0 Hz) power density in the EEG spectrum during non-REM sleep (data not shown). PLC-β4−/− mice, however, exhibited arrhythmic REM sleep occurrences throughout their circadian cycle (Fig. 1A). Although the length of daytime REM sleep in PLC-β4−/− mice (51.1±4.7 minutes, n = 6) was not significantly different from that in wild-type mice (63.6±6.0 minutes, n = 6, n.s. by Student's *t*-test), the length of nighttime REM sleep in PLC-β4−/− mice (46.7±6.9 minutes, n = 6) was increased 80% over that in wild-type mice (25.3±3.2 minutes, n = 6, *P*<.01 by Student's *t*-test). Also, in PLC-β4−/− mice, episodes of REM sleep interrupted by wakefulness frequently were immediately followed by another REM episode, creating an unusual sleep sequence with REM-sleep/wakefulness repeats (Fig. 1B,C). Single short transitions from wakefulness to REM sleep were observed both in the wild type (2.7±0.4 times for 24 hours, n = 6) and PLC-β4−/− mice (3.5±0.5 times for 24 hours, n = 6, n.s. by Student's *t*-test). Sets of more than 3 REM sleep/wakefulness repeats (Fig. 1B), however, occurred only in PLC-β4−/− mice both during the day (6.0±0.6 sets, n = 6) and the night (6.5±1.5 sets, n = 6). These results clearly demonstrate that the mechanism underlying the progression of the proper sleep sequence is impaired in PLC-β4−/− mice.

### Reduced Ultradian Body Temperature Rhythms in PLC-β4−/− Mice

**Figure 2:**
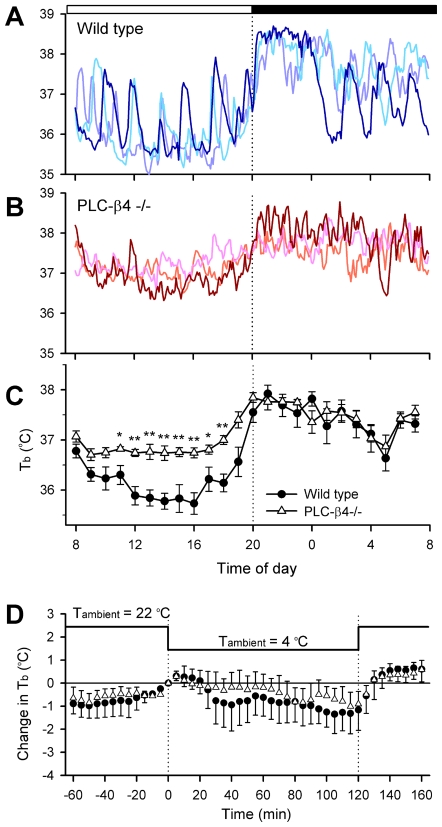
Reduced ultradian body temperature rhythms in PLC-β4−/− mice. Abdominal temperatures were measured in wild-type mice (A) and PLC-β4−/− mice (B) using a telemetry system. Representative abdominal temperature rhythms in 3 animals were plotted for each genotype. Although circadian rhythms of the temperature rhythm were manifest in both the wild-type and knockout mice, ultradian (i.e., 2–3 hour) oscillations were reduced in the knockout mice. White and black bars at the top denote light and dark periods. C. The temperature rhythm (±S.E.M.) was calculated for wild-type and PLC-β4−/− mice (n = 5 each). The daytime body temperature was significantly higher in the knockout mice than in the wild-type mice. *P<.05; **P<.01 compared with the corresponding wild-type group by Student's *t*-test. D. The ambient temperature was reduced from 22°C to 4°C for 2 hours to analyze the homeostatic thermal regulation in mice. No significant differences in the adaptive responses were found between the wild-type (n = 4, closed circle) and knockout (n = 4, open triangle) mice.

Since the sleep-wake cycles generally couple to body temperature rhythms, we next analyzed abdominal temperature rhythms in PLC-β4−/− mice. Regardless of the genotypes, circadian rhythms in abdominal temperature levels were synchronized to light-dark cycles being high during the nighttime and low during the daytime (Fig. 2A–C). Although this circadian synchronization is intact in the PLC-β4−/− mice, daytime temperature levels were significantly higher in PLC-β4−/− mice (36.9±0.1°C, n = 5) than that in wild type mice (36.0±0.1°C, n = 5, *P*<.01 by Student's t-test). The elevation in abdominal temperature levels depended on the circadian phase, since nighttime temperature levels were not different between PLC-β4−/− (37.3±0.1°C, n = 5) and wild type (37.4±0.1°C, n = 5). Coupling to the elevation of daytime temperature levels, ultradian (i.e., 2–3 hour periodic) temperature rhythms, which are observed frequently in the wild type mice, were significantly suppressed in PLC-β4−/− mice (Fig. 2A). To further analyze possible involvement of PLC-β4 in the thermoregulatory systems, mice were exposed to reduced (4°C) ambient temperature levels for 2 hours during the daytime. However, both genotypes displayed normal homeothermal response against shifts of ambient temperature (maximal temperature reduction = −1.5±0.8°C for PLC-β4−/− mice and −1.1±0.7°C for the wild type mice; n = 4 for both genotypes; n.s. by Student's *t*-test; Fig. 2D).

### Localization of PLC-β4 in the Brain

**Figure 3:**
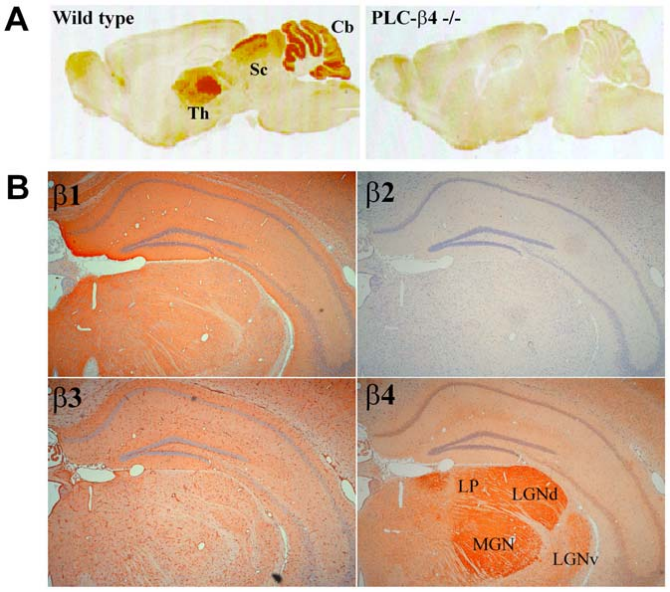
Localization of PLC-β families in the brain. A. Immunostaining with an anti-PLC-β4 antibody in sagittal brain sections from representative wild-type (left) and PLC-β4−/− (right) mice. Robust staining was observed in the thalamus (Th), superior colliculus (Sc), and cerebellum (Cb) in wild-type mice, but not in PLC-β4−/− mice. B. Immunostaining (orange-brown) of the thalamic geniculate nucleus and hippocampus of a wild-type mouse with PLC-β1-4 antibodies. No immunoreactivity was found for PLC-β2 and the section exhibits only cells counter-stained with cresyl violet (blue). Robust immunoreactivity for PLC-β4 was observed in the medial geniculate nucleus (MGN) and the dorsal lateral geniculate nucleus (LGNd). The ventral lateral geniculate nucleus (LGNv) exhibited relatively less immunoreactivity. DG, dentate gyrus; CA3, CA3 region of the hippocampus; PT, pretectal nucleus.

The immunohistochemistry was used to analyze the expression of PLC-β4 in the mouse brain and confirmed successful gene knockout in the mice (Fig. 3A). While PLC-β1 and –β3 were expressed throughout the brain (Fig. 3B), PLC-β4 was predominately expressed in the cerebellum, thalamic geniculate nucleus, lateral posterior nucleus (LP), and superior colliculus (Sc) (Fig. 3A,B). Among these loci, the thalamic geniculate nucleus had one of the most robust expressions of PLC-β4, especially in the medial geniculate nucleus (MGN) and LGNd (Fig. 3B).

### PLC-β4 Primarily Couples Group-1 mGluR in the LGNd

**Figure 4:**
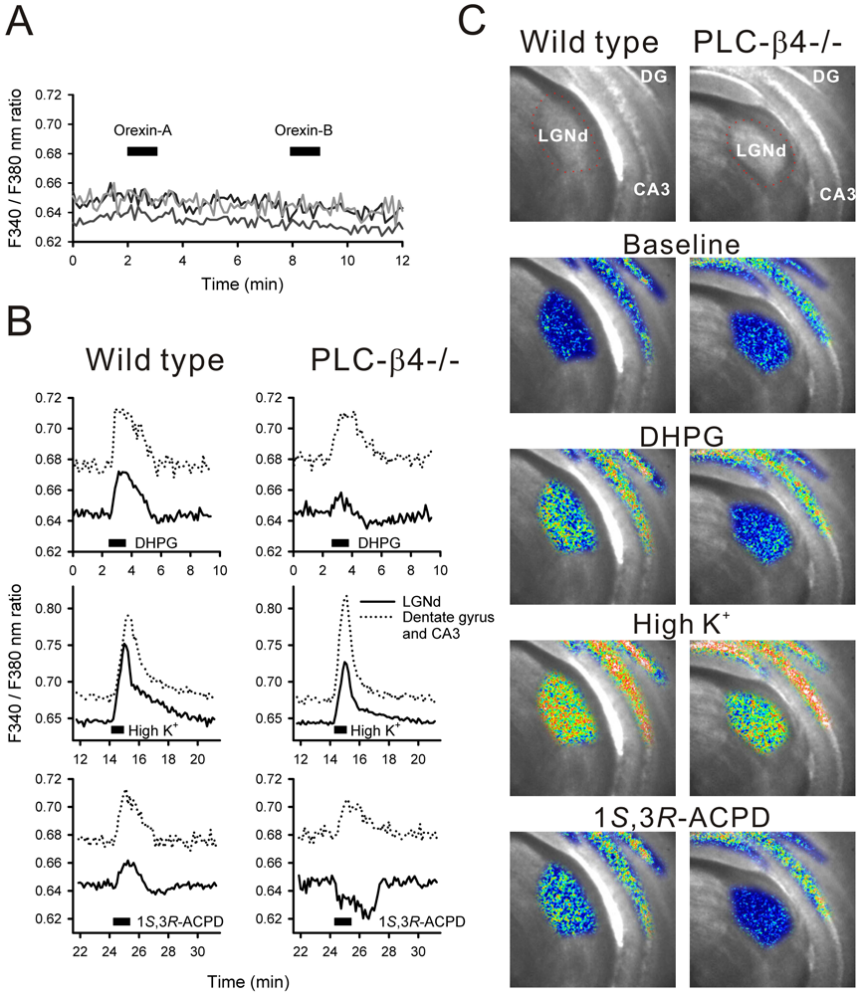
The metabotropic receptors linked to PLC-β4 in the LGNd were analyzed using Ca^2+^ imaging techniques. A. Orexin-A (300 nM) and orexin-B (300 nM) failed to mobilize intracellular Ca^2+^ in the LGNd of wild-type mice. Intracellular Ca^2+^ levels in LGNd from 3 different animals were plotted in this graph. B. The group-1 mGluR agonist, DHPG (100 µM), and the nonspecific mGluR agonist, 1*S*,3*R*-ACPD (100 µM), induced increased the intracellular Ca2+ in the LGNd (solid lines) of wild-type mice (left column). In PLC-β4−/− mice (right column), DHPG induced a significantly smaller Ca^2+^ increase while 1*S*,3*R*-ACPD decreased in Ca^2+^ in the LGNd. Control responses to 50 mM high-K^+^ stimulation (middle row) and in the dentate gyrus/CA3 region of hippocampus (broken lines) are also shown. C. Corresponding virtual color images of intracellular Ca^2+^ levels in the LGNd (approximate area is outlined in red in the top images) and dentate gyrus (DG)/CA3 region of the hippocampus were superimposed onto the transmitted light images shown at the top. Increasingly warmer colors indicate higher Ca^2+^ levels. Baseline was taken from the first frame of experiments. Frames of DHPG, high K^+^, and 1*S*,3*R*-ACPD were obtained from the peak Ca^2+^ response times in the dentate gyrus/CA3 region. All experiments were conducted in the presence of 1 µM tetrodotoxin.

We first examined the effects of orexin-A (300 nM) and –B (300 nM). However, no intracellular Ca^2+^ was mobilized in response to activation of either orexin receptor type in any LGNd slice examined (number of slices = 12; Fig. 4A). Also, application of either the muscarinic acetylcholine receptor agonist, McNA343 (100 µM), or the H_1_ histamine receptor agonist, HTMT (100 µM), failed to mobilize detectable intracellular Ca^2+^ in the LGNd (data not shown).

On the other hand, the group-1 mGluR agonist, DHPG (100 µM) significantly increased intracellular Ca^2+^ in all LGNd slices examined (number of slices = 6; Fig. 4B,C). DHPG produced significantly smaller Ca^2+^ increases (38.5% of the response in wild-type mice; *P*<.01 by Student's *t*-test) in the LGNd of PLC-β4−/− mice (Fig. 4B,C). DHPG also induced a slight decrease in Ca^2+^ after the increase in Ca^2+^ (Fig. 4B). The effects of the non-specific mGluR agonist, 1*S*,3*R*-ACPD (100 µM) were also examined to estimate the total mGluR response in the LGNd. In slices from wild-type mice, 1*S*,3*R*-ACPD produced a bi-phasic Ca^2+^ mobilization with a Ca^2+^ increase and subsequent Ca^2+^ decrease in the LGNd, whereas only the Ca^2+^ increase was observed in the dentate gyrus/CA3 region of the hippocampus (Fig. 4B,C). The Ca^2+^ increase in the LGNd was 39.9±3.8% smaller in amplitude than that in the dentate gyrus/CA3 region (number of slices = 6, *P*<.01 by Student's *t*-test). In slices from PLC-β4−/− mice, 1*S*,3*R*-ACPD produced only Ca^2+^ decreasing response in the LGNd, whereas the Ca^2+^ increasing response was intact in the dentate gyrus/CA3 region of the hippocampus (Fig. 4B,C).

### PLC-β4 Couples Group-1 mGluR Signaling Primarily in Relay Neurons Not in Interneurons

**Figure 5:**
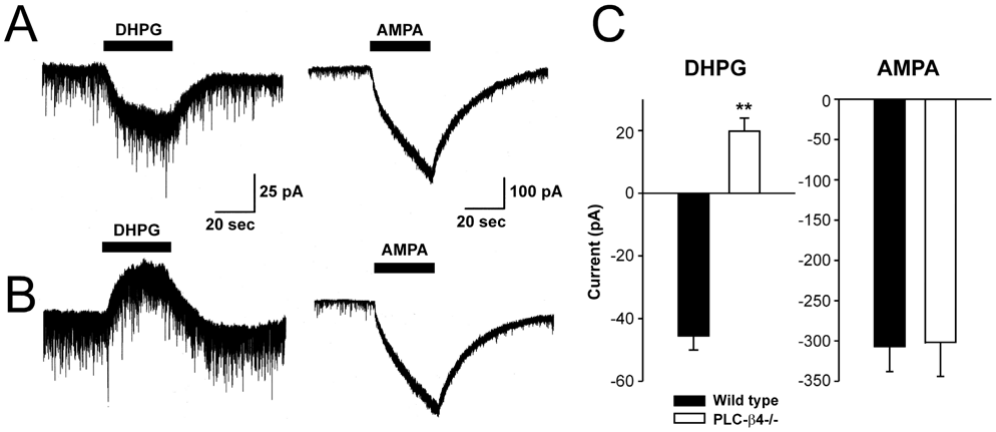
Responses of LGNd relay neurons to DHPG were examined using whole-cell voltage-clamp recordings. DHPG (200 µM) induced inward currents in LGNd relay-neurons in wild-type mice (A) but produced outward currents at the same holding membrane potential (−70 mV) in PLC-β4−/− mice (B). Stimulation of the same neurons with AMPA (10 µM) induced large inward currents in both wild-type (A) and PLC-β4−/− mice (B). C. The mean current amplitude was calculated and compared (error bars denote S.E.M.) n = 13–15 for each group. **P<0.01 compared with the wild-type group by Student's *t*-test. All experiments were conducted in the presence of 1 µM tetrodotoxin.

To examine single-cellular group-1 mGluR responses in the LGNd, we recorded activity in the relay neurons and the interneurons separately using whole-cell patch clamp (Fig. 5). DHPG-induced inward currents in interneurons tended to be smaller in PLC-β4−/− mice (12.3±2.5 pA, n = 11) than those in wild-type mice (22.6±5.6 pA, n = 7), but the difference was not statistically significant (*P* = .07 by Student's *t*-test). On the other hand, DHPG induced outward currents in relay neurons in PLC-β4−/− mice, whereas AMPA induced inward currents similar in both genotypes.

## Discussion

The results of the present study demonstrate that PLC-β4−/− mice exhibited increased REM sleep during the night and unusual wake-to-REM sleep transitions. Immunoreactivity to PLC-β4 was detected in the thalamus, superior colliculus, and cerebellum in mouse brain, with the highest expression levels in the thalamus observed in the LGNd and MGN (Fig. 3). To our knowledge, there is currently no evidence that suggests that either the cerebellum or MGN are involved in sleep regulation. Based on aspiration lesion data, Miller and colleagues suggested that the superior-colliculus-pretectum complex was involved in light-regulated REM-sleep episodes [25], but their more recent studies using more specific lesions demonstrated that it is the pretectum-geniculate complex that is important in this sleep regulation [26]. LGNd relay neurons are part of a glutamatergic corticothalamic feedback loop, which is hypothesized to have a critical role in the transition and maintenance of sleep stages [17]–[19]. Therefore, of the PLC-β4 immunoreactive brain areas, the LGNd is the most likely site of functional change underlying the irregular REM sleep observed in PLC-β4−/− mice.

Unusual body temperature rhythms were also observed in PLC-β4−/− mice (Fig. 2A). Body temperature rhythms in PLC-β4−/− mice displayed significantly smaller circadian amplitude with higher temperature levels during the daytime. This change in body temperature rhythms may not be due to the irregular set-point of thermoregulatory systems, since homeothermal responses against abrupt shift of ambient temperature levels were intact in PLC-β4−/− mice (Fig. 2B). It is rather conceivable that fragmentation of sleep episodes may be involved in this temperature elevation, since sleep durations are negatively correlated with body temperature levels in rodents [27]. In fact, ultradian rhythms in body temperature levels, which are generally coupled to short-term sleep-wake cycles [27], were almost absent in PLC-β4−/− mice (Fig. 2A). Recent immunohistochemical studies demonstrated that number of PLC-β4-immunoreactive cells displayed circadian rhythms in the suprachiasmatic nucleus, suggesting possible roles of PLC-β4 on circadian clock functions [28]. It has been shown that outputs from the SCN strongly influence REM sleep transitions [29], [30]. Therefore, reduced circadian amplitudes in body temperature rhythms may also be related to the outputs of circadian oscillator and resultant changes in REM sleep architectures, although neither our present results (Fig. 2A) nor previous studies [22] observed irregular circadian clock works, regarding the synchronization to environmental light-dark cycles.

In general, to determine the precise site of gene knockout effects on behavioral phenotypes, conditional knockout techniques and/or rescue techniques will be required. However, since PLC-β4 immunoreactivities were concentrated within the specific brain areas, we further analyzed the function of the LGNd, where is the most likely site of functional change underlying the irregular sleep sequence in PLC-β4−/− mice. The LGNd contains neurons that express various metabotropic receptors that may be involved in regulating sleep episodes [18]. To determine the predominant type of metabotropic receptors coupled to PLC-β4 in the mouse LGNd, we used macroscopic fura-2 Ca^2+^ imaging techniques. We first examined effects of orexin-A and –B on intracellular Ca^2+^ levels in the LGNd but failed to observe these responses (Fig. 4A). These results are consistent with previous reports showing little orexin immunoreactive projections to the LGNd [11] and no effects of orexin-A or –B on action potentials recorded in LGNd neurons [10]. In contrast, both DHPG and 1*S*,3*R*-ACPD increased intracellular Ca^2+^ in the LGNd (Fig. 4B,C). This result is consistent with the dense mGluR1a immunoreactivity previously observed in the LGNd [31]. Since PLC-β4−/− significantly reduced DHPG-induced Ca^2+^ response in the LGNd, it seems likely that PLC-β4 couples primarily to group-1 mGluR in the LGNd. The LGNd also contains other mGluR subtypes, possibly group-2 (i.e., G_i/o_-coupled inhibitory) mGluR [32]. Consistent with the presence of other mGluR subtypes, we observed a biphasic Ca^2+^ response followed by 1*S*,3*R*-ACPD stimulation and the negative phase was apparent when 1*S*,3*R*-ACPD was applied to the LGNd of PLC-β4−/− mice (Fig. 4B,C). Thus, the lack of PLC-β4 may result in a directional change in the response, from increasing Ca^2+^ to decreasing Ca^2+^, with simultaneous stimulation of all mGluR subtypes in the LGNd.

It is notable that PLC-β4−/− did not completely abolish group-1 mGluR-mediated cellular response in the LGNd. Previous studies have shown that mGluR activation in the cerebellum of PLC-β4−/− mice resulted in a decrease of only 27% in total phosphoinositide hydrosis [33]. Single-cell-based analysis of Purkinje cells demonstrated no measurable group-1 mGluR responses in lobe 6, but intact responses in lobe 9 of the cerebellum of PLC-β4−/− mice [13], [14], suggesting that there is heterogeneous expression of PLC subtypes in individual cells even within the same neuronal cluster. Also, group-1 mGluR are coupled to various intracellular signaling cascades, including PLC-independent cascades, such as G-protein activation of potassium channels in superior colliculus neurons [34]. Therefore, the residual Ca^2+^ response to group-1 mGluR activation in PLC-β4−/− mice observed in the present study may be explained by diversity in the cell types in the LGNd and intracellular signaling mechanisms linked to group-1 mGluR.

In fact, our voltage-clamp recordings demonstrated that PLC-β4−/− had small effects on LGNd interneurons whereas the DHPG-induced current had a reversed polarity in LGNd relay neurons. This observation indicates that (i) group-1 mGluR were coupled to other PLC subtypes in LGNd interneurons, (ii) PLC-β4 was critical for normal group-1 mGluR signaling in LGNd relay neurons and there was no compensatory coupling to other PLC subtypes in these neurons and (iii) group-1 mGluR activated PLC-independent signaling pathways in the relay neurons of PLC-β4−/− mice, presumably via G-protein activation of potassium channels [34], which, in wild-type mice was usually masked by phosphatidyl inositol cascades. Thus, disrupted group-1 mGluR signaling in LGNd relay neurons likely underlies the irregular sleep sequences in PLC-β4−/− mice, although further studies will be required to prove this hypothesis.

The molecular mechanisms and critical neuronal networks underlying REM sleep switching remain unclear. For example, gene-knockout mice lacking serotonin 1B receptors [35], norepinephrine [36], [37], or histidine decarboxylase (i.e., the histamine synthetic enzyme) [38] have been used to investigate REM sleep, but these knockout animals exhibit only a partial decrease or increase in REM sleep, and the phenotype is less dramatic than that of orexin-deficient or PLC-β4−/− mice. It is also unclear if the disruption of REM sleep in serotonin-, norepinephrine-, or histamine-deficient animals involves any changes in the hypothesized REM-sleep-regulating networks, because these receptors and substances are widely distributed throughout the brain, as in the case of orexin-positive fibers.

Tonegawa's group used the gene promoter sequence for the K_v_3.2 potassium channel to enable conditional knockout of T-type Ca^2+^ channels (Ca_v_3.1) in the rostral-midline thalamus, and observed frequent transitions between non-REM sleep and wakefulness [39]. Several nuclei in the rostral-midline thalamus have been shown to contain orexin-receptive neurons [9], [10]. Although REM sleep is intact in Ca_v_3.1 knockout mice, it is a reasonable hypothesis that heterogeneous neuronal clusters in the thalamus have a critical role in the regulation of sleep-wake transitions. The rostral-midline thalamus is near to but apparently separate from the PLC-β4-positive thalamic nuclei. Since the present study did not find direct molecular communications between orexin receptors and PLC-β4 in the LGNd, neuronal network communications among the LGNd, the rostral-midline thalamus, and/or other orexin-positive nuclei thus appear to have a role in regulation of sleep stages and sequences.

In conclusion, the present results demonstrate that PLC-β4−/− mice display fragmented sleep with unusual wake-to-REM sleep transitions, both during the day and nighttime. Also, PLC-β4−/− mice reduced ultradian body temperature rhythms and elevated body temperatures during the daytime. Although critical site(s) of PLC-β4−/− actions to produce these behavioral phenotypes are still remaining to be determined, localized expression of PLC-β4 in the brain lead us to propose that the most likely site is the LGNd. In fact, we demonstrated disrupted group-1 mGluR signaling in LGNd relay neurons in the PLC-β4−/− mice. Thus, the present results support the suggested thalamic function for the transition and maintenance of sleep stages.
